# Action of Different Medicines on the Mental Faculties

**Published:** 1861-05

**Authors:** 


Actions of Different Medicines on the Mental Faculties.—By *Professor Otto.*—All stimulant and exciting medicines increase the quantity of blood sent to the brain. If this quantity exceeds a certain amount, then most of the faculties of the mind become over-excited. Nevertheless the degree of this action is observed to vary a good deal in different cerebral organizations; and it is also found that certain stimulants exercise a peculiar and characteristic influence upon special or individual faculties. Thus ammonia and its preparations, as well as musk, castor, wine, and ether, unquestionably enliven the imaginative powers, and thus serve to render the mind more fertile and creative. The empyreumatic oils are apt to induce a tendency to melancholy and mental hallucinations. Phosphorus acts on the instinct of propagation, and increases sexual desire; hence, it has often been recommended in cases of impotence. Iodine seems to have a somewhat analogous influence, but then it often diminishes, at the same time, the energy of the intellectual powers. Cantharides, it is well known, are a direct stimulant to the sexual organs; while camphor tends to moderate and lull the irritability of these parts.

Of the metals, arsenic has a tendency to induce lowness and depression of spirits; while the preparations of gold serve to elevate and excite them. Mercury is exceedingly apt to bring on a morbid sensibility, and an inaptitude for all active occupation.

Of narcotics, opium is found to augment the erratic propensities, as well as the general powers of the intellect, but more especially the imagination. Those who take it in excess are, it is well known, liable to priapism. In smaller doses it enlivens the ideas and induces various hallucinations; so that it may be truly said that, during the stupor which it induces, the mind continues to be awake while the body is asleep. In some persons opium excites inordinate loquacity. Dr. Gregory says that this effect is observed more especially after the use of the muriate of morphia. He noticed this effect in numerous patients, and he then tried the experiment on himself with a similar result. He felt, he tells us, while under the operation, an invincible desire to speak, and possessed, moreover, an unusual fluency of language. Hence he recommends its use to those who may be called upon to address any public assembly, and who have not sufficient confidence in their own unassisted powers.

Other narcotics are observed to act very differently on the brain and its faculties from opium. Belladona usually impairs the intellectual energies; hyoscyamus renders the person violent, impetuous and ill-mannered; conium dulls and deadens the intellect, and digitalis is decidedly antiaphrodisiac. Hemp will often induce an inextinguishable gaiety of spirits; it enters into the composition of the intoxicating drink which the Indians call *bauss*. The use of *amanita muscaria* is said to have inspired the Scandinavian warriors with a wild and ferocious courage. Tobacco acts in a very similar manner with opium, even in those persons who are accustomed to its use; almost all smokers assert that it stimulates the powers of the imagination.

If the psychological action of medicines were better known, medical men might be able to vary their exhibition, according to the characters and mental peculiarities of their patients. The treatment of different kinds of monomaniacal derangement also might be much improved, and it is not improbable but that even a favorable change might be wrought on certain vicious and perverse dispositions, which unfortunately resist all attempts at reformation, whether in the way of admonition, reproof, or even of correction.

